# Microstructure and Properties of Atmospheric Plasma Sprayed (Al,Cr)_2_O_3_–TiO_2_ Coatings from Blends

**DOI:** 10.1007/s11666-021-01289-6

**Published:** 2021-11-30

**Authors:** Maximilian Grimm, Susan Conze, Lutz-Michael Berger, Rico Drehmann, Thomas Lampke

**Affiliations:** 1 Materials and Surface Engineering Group, Institute of Materials Science and Engineering, Chemnitz University of Technology, D-09107 Chemnitz, Germany; 2 Fraunhofer IKTS, Fraunhofer Institute for Ceramic Technologies and Systems, D-01277 Dresden, Germany

**Keywords:** alumina, atmospheric plasma spray (APS), ceramic coating, chromia, titania, properties, ternary system

## Abstract

Coatings prepared from chromia-rich (Al,Cr)_2_O_3_ solid solution (ss) feedstock powders are intended to improve the properties of Cr_2_O_3_ coatings, but are rarely studied so far. In this work, the processability of a commercial (Al,Cr)_2_O_3_ solid solution (ss) powder containing 78 wt.% Cr_2_O_3_ by atmospheric plasma spraying (APS), the corresponding coating microstructures and properties were investigated. Possible further improvements were expected by blending with 2, 23 and 54 wt.% TiO_*x*_ powder. For comparison, plain Cr_2_O_3_ and TiO_*x*_ coatings were studied as well. The microstructures were analyzed using SEM, EDS and XRD measurements. Hardness (HV0.3) was measured, as well as the dry unidirectional sliding wear resistance and the abrasion wear resistance (ASTM G65). Moreover, the corrosion and electrical insulating properties were measured. The (Al,Cr)_2_O_3_ ss showed only a small change of the composition, and the formation of *γ*-Al_2_O_3_, as found for alumina-rich (Al,Cr)_2_O_3_ ss powders, was avoided. Compared to the plain chromia coating, some improvements of the processability and coating properties for the ss (Al,Cr)_2_O_3_ coating were found. The most balanced coating performance was achieved by blending the ss (Al,Cr)_2_O_3_ with 2 wt.% TiO_*x*_, as this coating showed both a high sliding and abrasion wear resistance, in combination with a high corrosion resistance.

## Introduction

Thermally sprayed Cr_2_O_3_ coatings are widely used due to their excellent wear and corrosion properties (Ref 1-3). The main application is anilox rolls for printing machinery (Ref 4) where the good patterning by laser is a further advantage.

However, manufacturing of chromium oxide coatings meets some difficulties. Chromium oxide feedstock powders may contain impurities of metallic chromium, which is in particular detrimental in the laser engraving process of the corresponding coatings. Advantageously, Cr_2_O_3_ does not show any phase transformation during spraying, but has a very high melting temperature of over 2300 °C and shows low deposition efficiencies of about 40%, maximum 60% in the case of APS (Ref 2). Bolelli et al. (Ref 1) indicate that Cr_2_O_3_ volatilizes at high temperature in both reducing and oxidizing environments, and in both cases, this occurs well below its melting point. A reduction in the reducing part of the plasma can be responsible for the change of color from green to black due to a small oxygen deficiency in Cr_2_O_3_ (Ref 5) or, in more severe cases, for formation of metallic chromium (Ref 1). However, most often oxidation of Cr_2_O_3_ and formation of volatile CrO_3_ is discussed (Ref 1, 3, 6). In result of cooling, an immediate reconversion occurs and Cr(VI) is very rarely detectable in the coatings. However, its possible occurrence represents a risk for health and environment. In order to meet these challenges, Cr-rich (Al,Cr)_2_O_3_ solid solution feedstock powders were developed (Ref 7).

Al_2_O_3_ and Cr_2_O_3_ have the same crystallographic corundum structure and form a (Al,Cr)_2_O_3_ solid solution over the entire concentration range of the phase diagram at high temperatures (Ref 8, 9). However, there is also a non-symmetrically located miscibility gap in the system with a maximum temperature of 1295 °C according to Besmann et al. (Ref 8). Previous research has focused on the use of Al_2_O_3_-rich (Al,Cr)_2_O_3_ solid solution feedstock powders. They are proved to be effective to suppress but not fully to avoid the *α*-Al_2_O_3_ *γ*-Al_2_O_3_ transformation when spraying Al_2_O_3_-rich compositions. However, the optimum chromia content is not known so far (Ref 2, 3, 9-11).

In general, there are only few studies on coating development for the Cr_2_O_3_-rich side of the binary Al_2_O_3_-Cr_2_O_3_ system. There are few works dealing with mechanical blends sprayed by detonation gun spraying (DGS) (Ref 12) and APS (Ref 13-15). Despite the presence of metallic chromium, APS and HVOF-sprayed coatings containing 75 wt.% Cr_2_O_3_ in the fused and crushed feedstock powders showed a better abrasion wear resistance and corrosion performance than coatings with lower Cr_2_O_3_ content (Ref 16). The most systematic study with two commercial powders was performed most recently by Bolelli et al. (Ref 1) with focus on the tribological characterization of APS coatings. Another study using a commercial solid solution feedstock powder of unknown origin should be mentioned (Ref 17).

There are indications in the literature that the coating properties of binary compositions can be further improved by the third oxide of the Al_2_O_3_-Cr_2_O_3_–TiO_2_ system (Ref 10, 18, 19), in particular additions of TiO_2_ to coatings of the binary Al_2_O_3_-Cr_2_O_3_ system. When spraying ternary blends of single oxide powders by APS, some incorporation of Ti into Cr_2_O_3_ splats (Ref 10, 18, 19) was observed. A homogeneous distribution of the metallic constituents was found in an APS coating from an experimental agglomerated and sintered feedstock powder, although in the feedstock powder, Ti was separated from the Al_2_O_3_-rich ss (Al,Cr)_2_O_3_. In another study, coatings prepared from blends of Al_2_O_3_-3%TiO_2_ with Cr_2_O_3_ are reported (Ref 20), thus in fact already belonging to the ternary Al_2_O_3_-Cr_2_O_3_–TiO_2_ system.

The aim of the current study was to investigate the improvement of the processing properties, coating microstructure, hardness, tribological, corrosion and electrical properties of APS Cr_2_O_3_-based coatings. For this, coatings were sprayed from a commercial (Al,Cr)_2_O_3_ solid solution powder, and blends of this powder with different amounts of a TiO_*x*_ powder. Plain Cr_2_O_3_ and TiO_*x*_ coatings were investigated for comparison. Thus, compared to earlier studies where ternary blends of single oxides were investigated (Ref 10, 18, 19), in this study, a binary blend is used, where two metallic elements (Al and Cr) are homogeneously distributed in one type of feedstock powder particles, where the third element (Ti) is contained in the other feedstock powder particles. This work presents part of the exploration of the chromia-rich corner of the ternary Al_2_O_3_-Cr_2_O_3_–TiO_2_ system aiming to develop improvements for single and binary oxide compositions.

## Materials and Methods

As this study is part of extensive investigations of APS coatings in the ternary system Al_2_O_3_-Cr_2_O_3_–TiO_2_, a uniform methodology of investigation of coating formation and characterization is applied (Ref 10, 18, 19).

The feedstock powders used in this study were Cr_2_O_3_, Al_2_O_3_-75%Cr_2_O_3_ (hereinafter referred to as AC75) and TiO_*x*_. Information on the manufacturer, the production process, particle size and granulometric data determined by laser diffraction analysis (Cilas 930, Cilas, Orléans, France) are listed in Table 1. Both single oxide feedstock powders have already been used in our earlier studies (Ref 18, 19). Due to the manufacturing process, the titania powder is non-stoichiometric and designated in the following as TiO_*x*_. According to the results of gravimetric measurements in an earlier study, *x* in TiO_*x*_ is about 1.9 (Ref 21) with an inhomogeneous distribution of oxygen in the powder particles. Different compositions of the commercial ss (Al,Cr)_2_O_3_ powder were used in a recent study by Bolelli et al. (Ref 1). In order to compensate the different thermophysical properties of AC75 and TiO_*x*_ powders in the blends, a smaller particle size fraction of the former was selected.

**Table 1:** Compilation of feedstock powders (f and c—fused and crushed, a and s—agglomerated and sintered)

Material	Supplier and Trade name	Manufacturing method	Particle size (µm)	Granulometric data		
				d_10_	d_50_	d_90_
Cr_2_O_3_	GTV, Germany 40.06.1	f and c	− 45 + 15	19	34	54
(Al,Cr)_2_O_3_	Saint Gobain Coating Solutions, France, Ruby VF	a and s	− 30 + 10	14	21	31
TiO_*x*_	Ceram Ingenieurkeramik, Germany, Rutil–TiO_2_	f and c	− 45 + 20	22	39	61

**Fig. 1:**
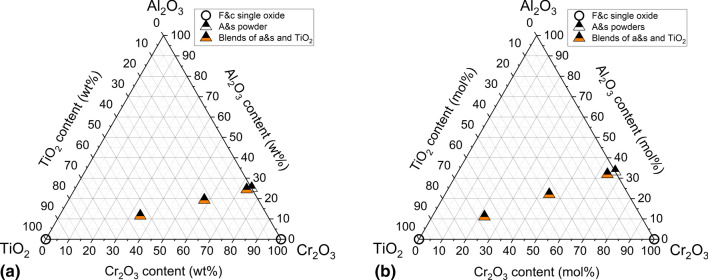
Feedstock powder compositions in the Al_2_O_3_-Cr_2_O_3_–TiO_2_ system in this study in (a) wt.% and (b) mol%

After drying, three blends were prepared using a tumbler mixer. Their composition and short designations are summarized in Table 2. The composition T2 was selected based on information in the literature on the positive effect of small TiO_2_ additions of < 3 wt.% in Al_2_O_3_-Cr_2_O_3_ ceramics leading to the increase in density and reduction of sintering temperature (Ref 22-24). Preliminary experiments with sintered bodies using spark plasma sintering (SPS) have confirmed this advantage. These experiments have also indicated on the possibility of formation of solid solutions (Al,Cr)_2_TiO_5_ and (Cr,Al)_2_TiO_7_ , for compositions T23 and T54, respectively. The positions of the feedstock powders and blends in the Al_2_O_3_-Cr_2_O_3_–TiO_2_ system are visualized in Fig. 1.

**Table 2:** Composition of the powder blends

Designation of Blend/coating	Content, wt.%	
	(Al,Cr)_2_O_3_	TiO_*x*_
AC75-T2	97.6	2.4
AC75-T23	77.0	23.0
AC75-T54	45.9	54.1

Feedstock powder morphologies and particle cross sections were investigated by scanning electron microscopy (SEM) (LEO 1455VP, Zeiss, Oberkochen, Germany) using an acceleration voltage of 25 kV. In addition, the phase composition was studied by x-ray diffraction (XRD) using a D8 Advance diffractometer (Bruker AXS, Billerica, MA, the USA) with a Bragg–Brentano geometry operating with Cu K_*α*_ radiation in a range of 2*θ* = 15-120° with a step size of 0.02° and 3 s/step. The composition of the AC75 powder was determined by the change of the lattice parameter between Cr_2_O_3_ (eskolaite) and *α*-Al_2_O_3_ (corundum) based on Vegard’s law. For the lattice parameter determination, the software TOPAS (Ref 25) with the Pawley fit algorithm (Ref 26) was used.

Low carbon steel (S235, 1.0038) was used as substrate material. To ensure good adhesion of the coating, directly before spraying, the substrates were grit-blasted with alumina (EK-F 24) (0.3 MPa, 20 mm distance, 70° angle) and cleaned in an ultrasonic ethanol bath. Coatings were deposited with an F6 APS torch (GTV, Luckenbach, Germany) using a single spray parameter set as given in Table 3. The powder feed rate by volume was constant for all materials, resulting in a minimum feed rate by weight for TiO_*x*_ and a maximum feed rate per weight for Cr_2_O_3_. The powder carrier gas (argon) flow rate has been adapted for each feedstock powder to ensure injection of the powder particles into the center of the plasma torch. It is proposed that this set of parameters largely avoids changes in the chemical composition due to the specific processing properties of the constituents of the powder blends. The substrate temperature was measured by a thermocouple placed just beneath the surface (<0.5 mm) in a hole drilled from the backside. Interruptions for cooling were made in order to keep the substrate temperature below 250 °C. The number of passes was adapted in a manner to reach the target coating thickness of 200-300 µm. After measurement of the coating thickness as indicated below, the average thickness of the layers was calculated using the number of passes. Due to the constant volumetric feed rate applied for all powders, the average thickness per layer can be used for estimation of the deposition efficiency.

**Table 3:** The spray parameters of the APS process (F6 torch), * for constant volume powder feed rate.

Flow rate Ar, l/min	41
Flow rate H_2_, l/min	11
Current, A	600
Spraying distance, mm	110
Traverse speed, m/s	0.4
Number of passes*	3 (TiO_*x*_)-8 (Cr_2_O_3_)
step increment, mm	5
Powder feed rate, g/min*	30 (TiO_*x*_)-40 (Cr_2_O_3_)

The cross sections of the coatings were prepared by standard metallographic procedures. An optical microscope GX51 (Olympus, Shinjuku, Japan) equipped with a SC50 camera (Olympus, Shinjuku, Japan) was used to investigate the microstructure of the coatings. Five images taken at a magnification of 200x were analyzed to evaluate the porosity using the image analysis method provided by the camera software. The micrographs were also used to determine the coating thickness at ten evenly distributed points of the cross section. Moreover, SEM examinations using the backscattered electron detector (BSD) were carried out for investigation of the coating microstructures using the same conditions as for the feedstock powders. The chemical composition of the coatings, both average and locally, was determined by EDS analysis (GENESIS, EDAX, Mahwah, NJ, the USA). To determine the average coating composition, large regions of the cross section (400 × 150 µm^2^) were measured at three randomly selected locations. The local chemical composition, e.g., of splats and inclusions, was studied by five measuring points. When positioning the measuring points, care was always taken to ensure that the influence of surrounding areas was minimized. The phase compositions were investigated from the XRD patterns measured with the same conditions as the feedstock powders.

The Vickers microhardness of the coatings was measured on the cross sections using a Wilson Tukon 1102 device (Buehler, Uzwil, Switzerland). Ten indentations with a test load of 2.94 N were examined.

The dry unidirectional sliding wear resistance of the coatings was investigated at room temperature using a ball on disk tribometer (Tetra, Ilmenau, Germany) and polished, rotating specimens with a surface roughness of Ra < 0.25 µm with the test parameters given in Table 4. For each coating composition, three runs were carried out. An optical 3D profilometer MikroCAS (LMI, Teltow, Germany) was used to determine the volume of the resulting wear marks.

**Table 4:** Sliding wear test parameters

Force	Radius	Speed	Cycles	Wear distance	Counter body	
					Material	Diameter
10 N	5 mm	0.05 m/s	15916	500 m	Al_2_O_3_	6 mm

The abrasion wear resistance of the coatings was investigated using the rubber wheel test according to ASTM G65-04. Due to the high wear rates, thicker coatings (~ 500 µm) were used. For each coating, two samples were used, performing three consecutive runs using test according to procedure E (load 130 N, distance 718 m). Due to the typical shape of the large worn area, a direct measurement of the volume using a profilometer is not possible. Thus, the mass loss of the samples was determined by weighing them before and after the test runs. For better comparability, the wear volume was calculated from the theoretical density of the coating material taking into account the porosity and phase composition.

For exposure testing with analogous test conditions as in the earlier studies (Ref 10, 18), stainless steel (alloy designation 1.4462, corresponds to X2CrNiMoN22-5-3) was selected as the substrate material in order to investigate the corrosion resistance of the coating material alone. The coated substrates were immersed in 1 N H_2_SO_4_ (corresponds to 0.5 M H_2_SO_4_) in isothermal conditions at 85 °C for 300 h in the same manner as in a previous work (Ref 10, 27). A special PTFE sample holder for coatings protected the substrate, so that only a defined sample surface of 1.19 cm^2^ was in contact with the medium. Continuous stirring ensured a homogeneous chemical environment for all samples. The mass loss was determined after 300 h and drying for 4 h at 110 °C. Two samples of each coating were tested.

The conditions of the electrical measurements also correspond to those of the earlier studies (Ref 10, 18). The DC resistivity of the coatings was determined using the four-point electrode method for a low specific resistivity ≤ 10^2^ Ω m, but for higher resistivity, a two-electrode arrangement was used. For the four-point method, the substrate was first coated with an insulating MgAl_2_O_4_ coating by APS with a thickness of 200 µm. Two strips with a size of 35 × 5 mm^2^ were deposited directly on the insulating coating using a mask. The specific thickness of the conductive strips and their width as well as the electrode distance were determined using an optical profilometer (FRT-MicroProf, Fritsch Research and Technology GmbH, Germany). The measurement was performed with a 100 W power supply (Keithley 2425 sourcemeter) and a multimeter (Keithley 2000, Keithley Instruments). A voltage of 2 V at room temperature was used. For the high-resistance coatings, a stainless steel plate with an area of 300 mm^2^ was used as the counter electrode and the coating as the working electrode in a two-electrode arrangement. To ensure good electrical contact to a stainless steel plate over the entire as-sprayed coating surface, a graphite fleece (Sigracell GFD 2.5EA, SGL Carbon SE, Wiesbaden, Germany) with the same geometry as the counter electrode was applied. The DC resistivity measurement for the high-resistance coatings was performed with resistance meter ADVANTEST R8340 at room temperature with a voltage of 100 V after 60 s once a stable current was obtained. A comparative measurement using a standard resistor with 10010^6 ^Ω was performed to control the test device. The four-point measurements reveal generally lower resistivity values caused by the higher contribution of the surface conductance in contrast to the two-electrode arrangement.

The dielectric breakdown strength (DBS) measurements were performed with a two-electrode arrangement with a stainless steel plate as the counter electrode and the coating as the working electrode. The coating was contacted with a soft conductive graphite fleece (Sigracell GFD 2.5EA, SGL Carbon SE) with a diameter of 5 mm, corresponding to a contact surface area of 15.7 mm^2^. The measurement was performed with a high voltage testing device (UG36, ETL Prüftechnik GmbH, Germany) with direct voltage increased with a rate of 0.1 kV s^-1^ up to maximum 6 kV according to DIN IEC 60672-2 (VDE 0335 Part 2) 10/2000 and DIN EN 60243-2 (VDE 0303, Part 22), 2014-08. Five measurements were carried out for each coating. The dielectric breakdown field strength *E*_*DBS*_ was calculated from the coating thickness *d* and the breakdown voltage *U* according to ${E}_{DBS}=\frac{U}{d}$.

## Results

**Fig. 2:**
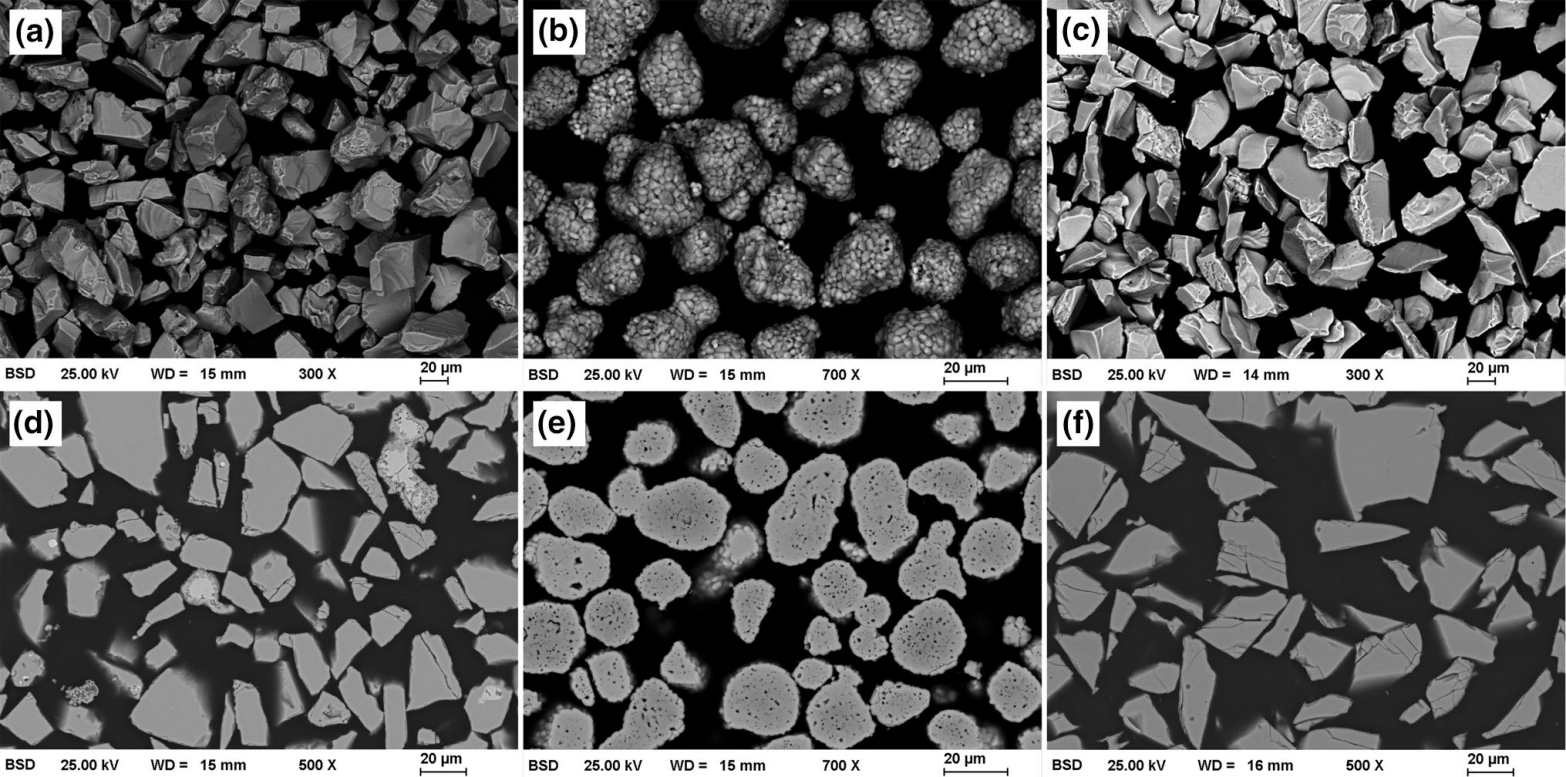
Morphologies and cross sections of the feedstock powders: (a, d) Cr_2_O_3_, (b, e) (Al,Cr)_2_O_3_, (c, f) TiO_*x*_

The SEM images, shown in Fig. 2(a-c), reveal the different morphologies of the feedstock powders resulting from the corresponding manufacturing routes. Both plain oxide feedstock powders present the typical angular morphology of powders produced by fusing and crushing. The AC75 powder particles show a largely spherical morphology consisting of a large number of small grains, typical for powders produced by agglomeration and sintering. Figure 2(d-f) shows cross sections of the powders. In the case of the chromium oxide powder, some particles show bright areas mainly in the cores of the particles. According to the EDS measurements, these are areas of a highly elevated chromium content. Some small differences in the grayscale levels also occur in the AC75 powder particles. According to the EDS measurements, slightly darker areas have a higher Al content. In addition, the particles show a high internal porosity. The particles of the titania powder show a uniform grayscale contrast.

**Fig. 3:**
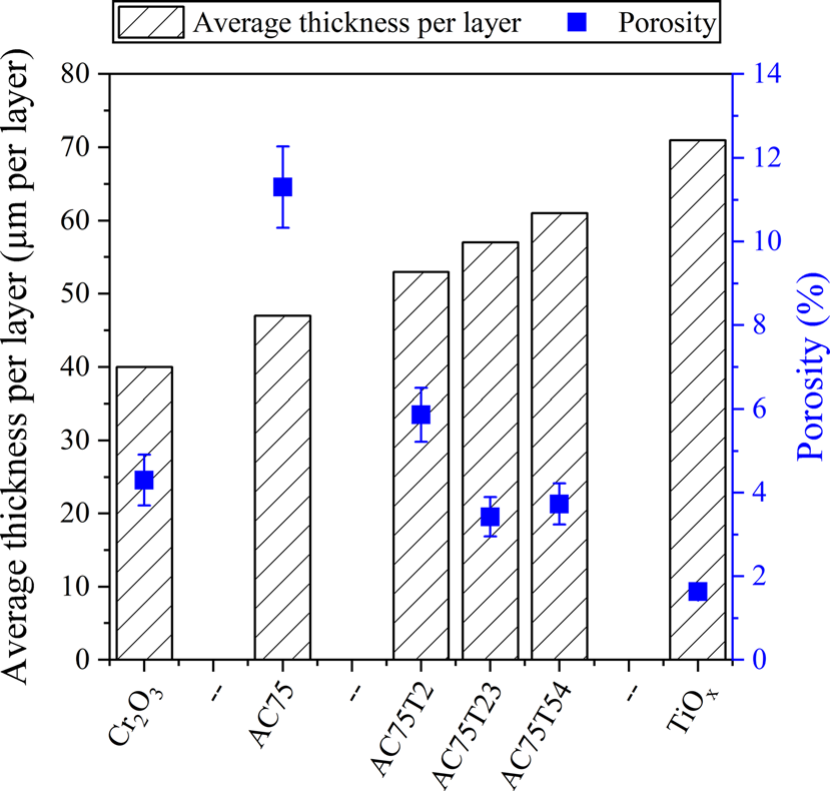
Average thickness per layer and porosity of the coatings

Figure 3 shows the average thickness per layer of the coatings. As expected, chromium oxide has the lowest thickness per layer (40 µm). For the AC75 powder, a higher thickness per layer of 47 µm was found (+ 17.5% compared to Cr_2_O_3_). For the powder blends, the average thickness per layer was further increased with the increasing amount of TiO_*x*_. The average thickness per layer of the plain TiO_*x*_ coating (71 µm) corresponds to an increase of 77.5% compared to the Cr_2_O_3_ coating. Figure 3 presents also the porosity of the coatings. Surprisingly, the porosity of the AC75 coating was much higher (> 10%) than the porosity of the plain chromium oxide coating (4.3%). The porosity of the plain TiO_*x*_ coating was 1.6% only. The coatings from the powder blends showed a significant reduction in porosity, even with the very low TiO_*x*_ content of 2%. However, the very low porosity of the plain TiO_*x*_ coating was not reached.

**Fig. 4:**
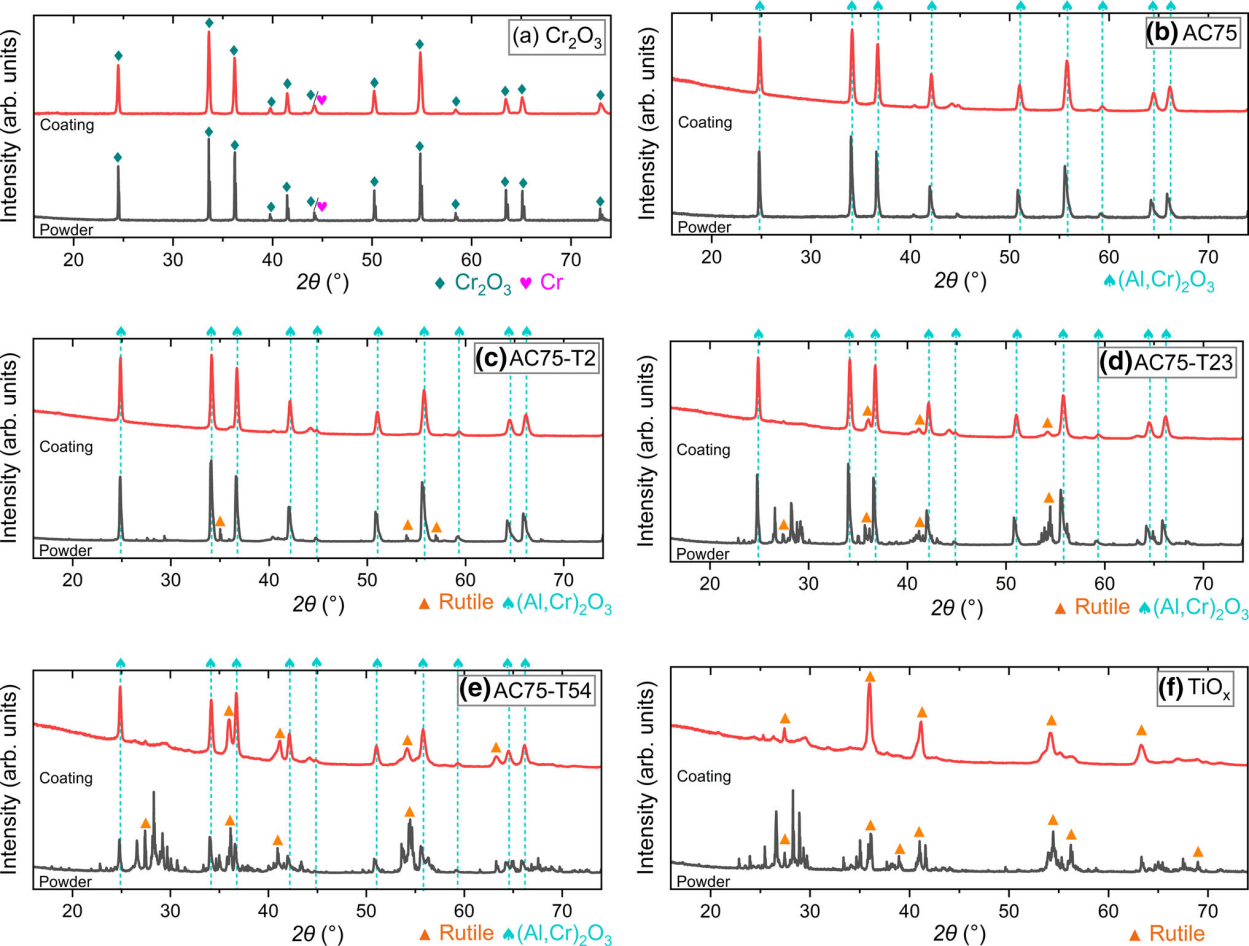
Diffraction patterns of the feedstock powders and corresponding coatings: (a) Cr_2_O_3_, (b) AC75, (c) AC75-T2, (d) AC75-T23, (e) AC75-T54, (f) TiO_*x*_. Unlabeled peaks in the patterns in Fig. 4c-f relate to non-stoichiometric titania phases, having partially coinciding peaks with rutile

The XRD patterns presented in Fig. 4 combine the feedstock powders (lower patterns) with those of the corresponding coatings (upper patterns). For chromium oxide in Fig. 4(a), no significant changes between the feedstock powder and the coating were found. Both the powder and the coating consist predominantly of eskolaite (PDF 00-038-1479) and contain small amounts of metallic chromium. The XRD pattern of the AC75 powder, displayed in Fig. 4(b), shows the peaks of the (Al,Cr)_2_O_3_ solid solution with positions between those of the α-Al_2_O_3_ (corundum, PDF 00-042-1468) and Cr_2_O_3_ (eskolaite, PDF 00-038-1479) standards. The refinement revealed a content of 78 wt.% (71 mol%) Cr_2_O_3_ according to Vegard’s law, corresponding to 12 at.% Al and 28 at.% Cr. This is in good agreement with the nominal composition and the information in the certificate of the manufacturer. Peaks of metallic chromium were not found. The XRD pattern of the AC75 coating shows a slight peak shift to higher diffraction angles, indicating a reduction of the Cr_2_O_3_ content in the (Al,Cr)_2_O_3_ from 78 wt.% in the powder to 72 wt.% (64 mol%) in the coating. This was also observed for the coatings sprayed from the blends. Some very small peaks could not clearly identified.

All XRD patterns of the coatings sprayed from the blends (Fig. 4c-e) present peaks of ss (Al,Cr)_2_O_3_ and TiO_*x*_ as well as very small peaks which could not clearly identified. This corresponds to the observations for the plain AC75 and TiO_*x*_ coatings. The intensity of the peaks related to TiO_*x*_ increases with increasing TiO_*x*_ content, while those of the ss (Al,Cr)_2_O_3_ peaks decrease simultaneously. Due to the small content of only 2.4 wt.% of TiO_*x*_ in the AC75-T2 coating, peaks of TiO_2_ or TiO_*x*_ are not observed. In the XRD patterns of the AC75-T23 and AC75-T54 coatings (Fig. 4d,e), the characteristic (110) peak of stoichiometric rutile is missing, while the (101) and (111) peaks, which are also characteristic for the non-stoichiometric phases, are present. The formation of new phases for the AC75-T23 and AC75-T54 coatings, as could be expected from the preliminary experiments, was not observed.

The XRD pattern of the plain titania powder in Fig. 4(f) shows the presence of non-stoichiometric TiO_*x*_ phases, together with some TiO_2_ indicated by the presence of the small (110) peak of rutile at 2*θ* = 27.4°. In the pattern of the coating, the (101) and (111) rutile peaks at 36.1° and at 41.2°, respectively, are most intense.

**Fig. 5:**
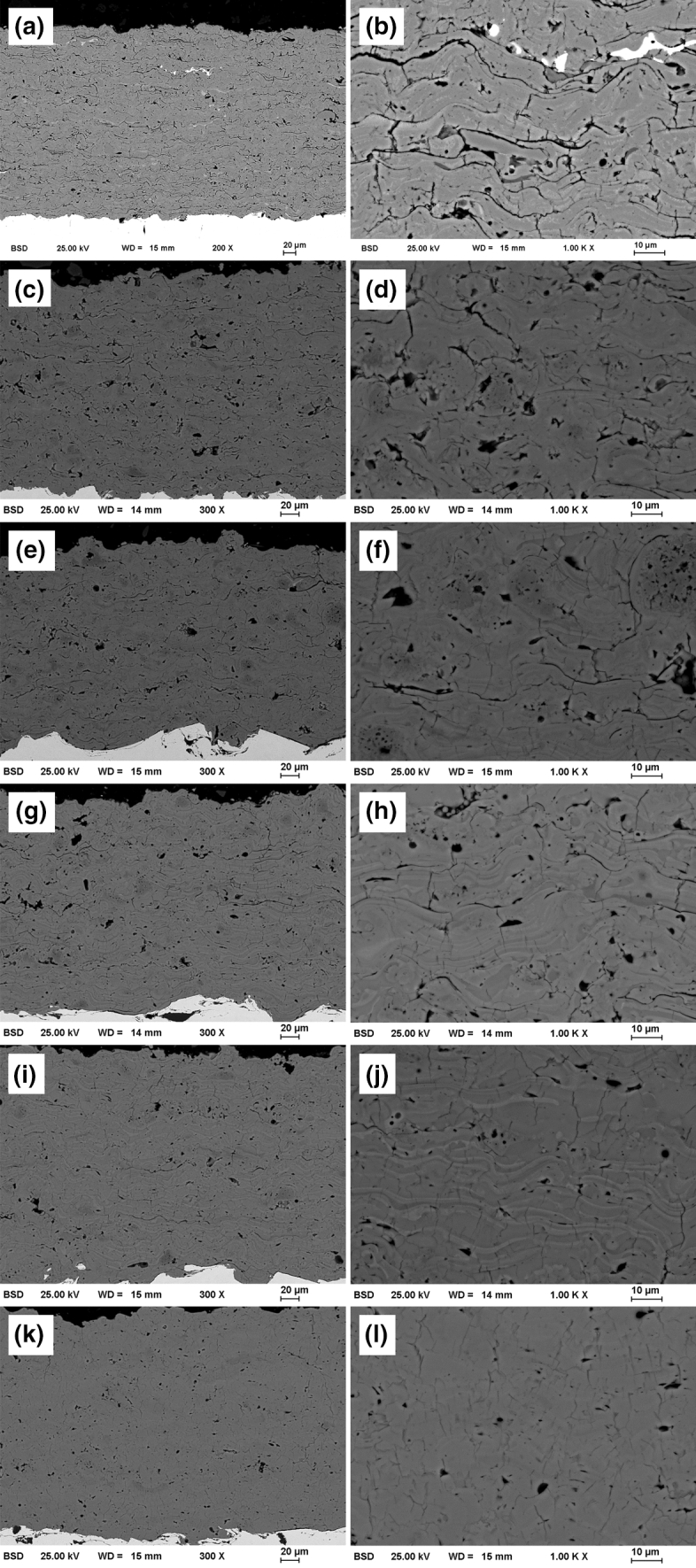
SEM cross section images of the coatings: (a,b) Cr_2_O_3_, (c,d) AC75, (e,f) AC75-T2, (g,h) AC75-T23, (i,j) AC75-T54, k,l) TiO_*x*_

The SEM images of the coatings are shown in Fig. 5. The chromia and AC75 coatings (Fig. 5c,d) have numerous pores and cracks; some severe cracks occur between individual splats. Few isolated very bright splats are found in the chromium oxide coating. In these areas, a very high chromium content (> 95%) is detected by EDX measurements. The AC75 coating shows small differences in the grayscale. In particular, non-melted particles appear darker (e.g., Fig. 5d,f). Like the AC75 coating, the coatings prepared from the powder blends (Fig. 5e-j) showed some minor variations in the grayscale levels. In addition, these coatings are characterized by a decreasing amount of pores and microcracks with increasing TiO_*x*_ content. The microstructure of the TiO_*x*_ coating (Fig. 5k,l) differs significantly. It shows a homogeneous grayscale with only few evenly distributed small pores and very small microcracks within the splats oriented vertically to the interface.

**Fig. 6:**
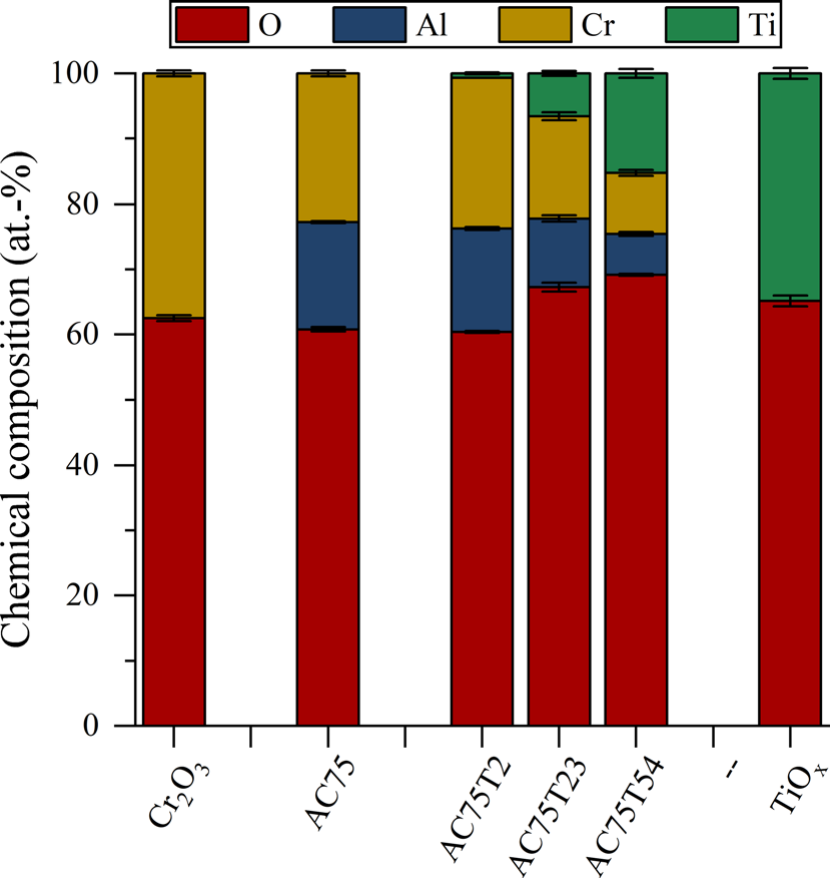
Average chemical composition of the coatings determined by EDS analysis

The average chemical compositions of the coatings determined by EDS are presented in Fig. 6. The coating compositions correspond to the initial spray powder compositions.

**Fig. 7:**
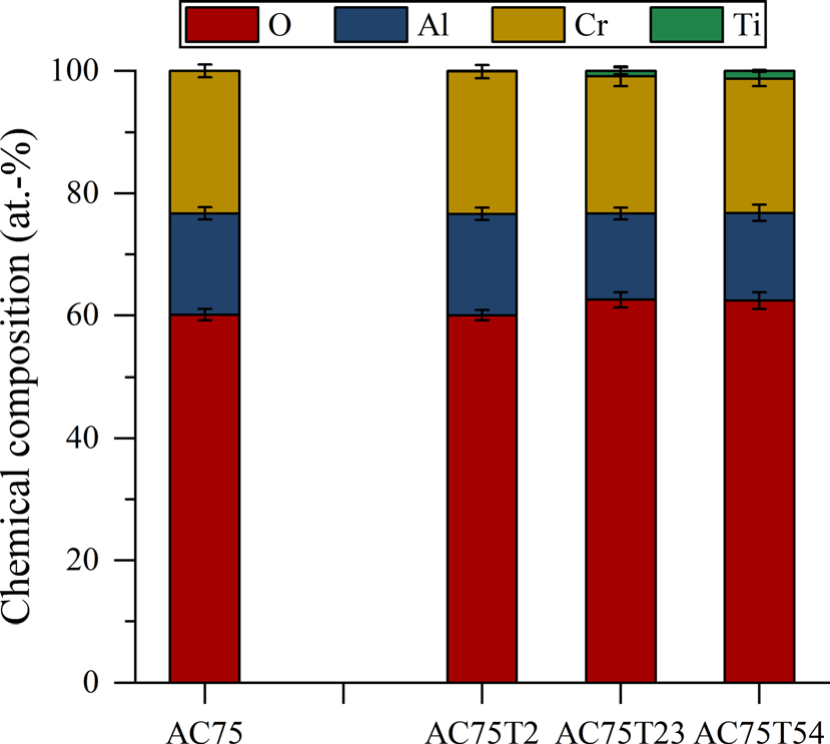
Average chemical composition of (Al,Cr)_2_O_3_ splats in the coatings determined by EDS analysis

Figure 7 shows the average elemental composition of the ss (Al,Cr)_2_O_3_ splats in the AC75 coating and in the coatings sprayed from the blends. While titanium was not detectable (< 0.1 at.%) in the ss (Al,Cr)_2_O_3_ splats of the AC75-T2 coating, in the AC75-T23 and AC75-T54 coatings, low contents of titanium (about 1.0 at.% and 1.7 at.%, respectively) are detected in these regions. The (Al,Cr)_2_O_3_ splats show different grayscales according to their individual composition. The amount of Al and Cr can vary by up to 3%. The brighter splats containing a higher amount of Cr also tend to contain a slightly higher amount of titanium. The amount of TiO_*x*_ splats in these coatings increases with the TiO_*x*_ content. These splats are visible as slightly brighter areas in the SEM images (e.g., Fig. 5j). The EDS point measurements did not reveal any remarkable differences in the chemical composition of the TiO_*x*_ splats. The oxygen content was found in the range 64-66 at.%, indicating non-stoichiometry.

**Fig. 8:**
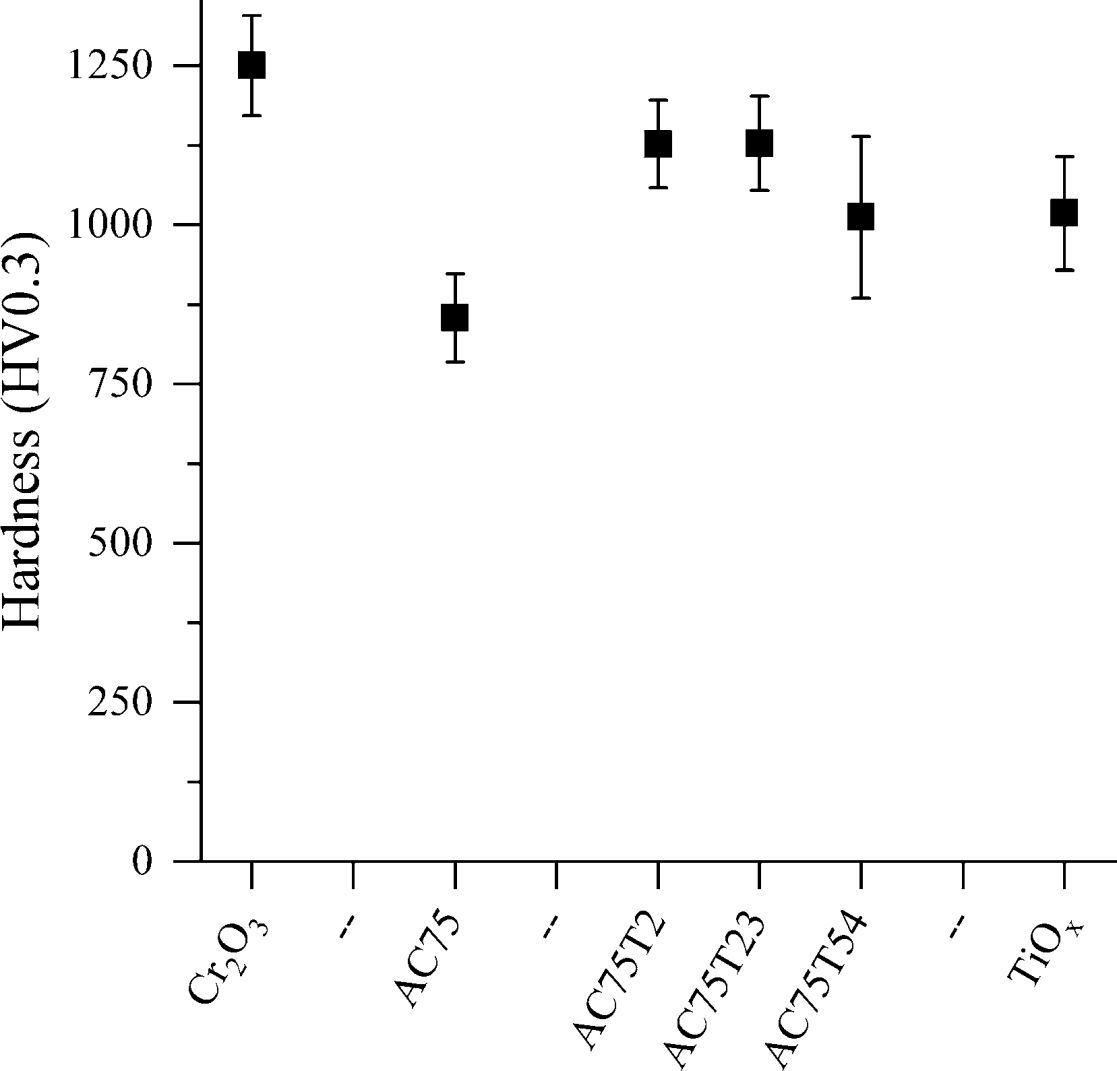
Hardness HV0.3 of the coatings

The results of the microhardness measurements, presented in Fig. 8, show that the Cr_2_O_3_ coating had the highest hardness (1250 ± 79 HV0.3). Surprisingly, the hardness of the AC75 coating was significantly lower (854 ±69 HV0.3) and even the lowest of all coatings in this study. The addition of TiO_*x*_ in coatings AC75-T2 and AC75-T23 increased the hardness significantly (1128 ± 74 HV0.3). For AC75-T54, the hardness decreased again, but was, however, similar to the plain TiO_*x*_ coating (1018 ± 89 HV0.3).

**Fig. 9:**
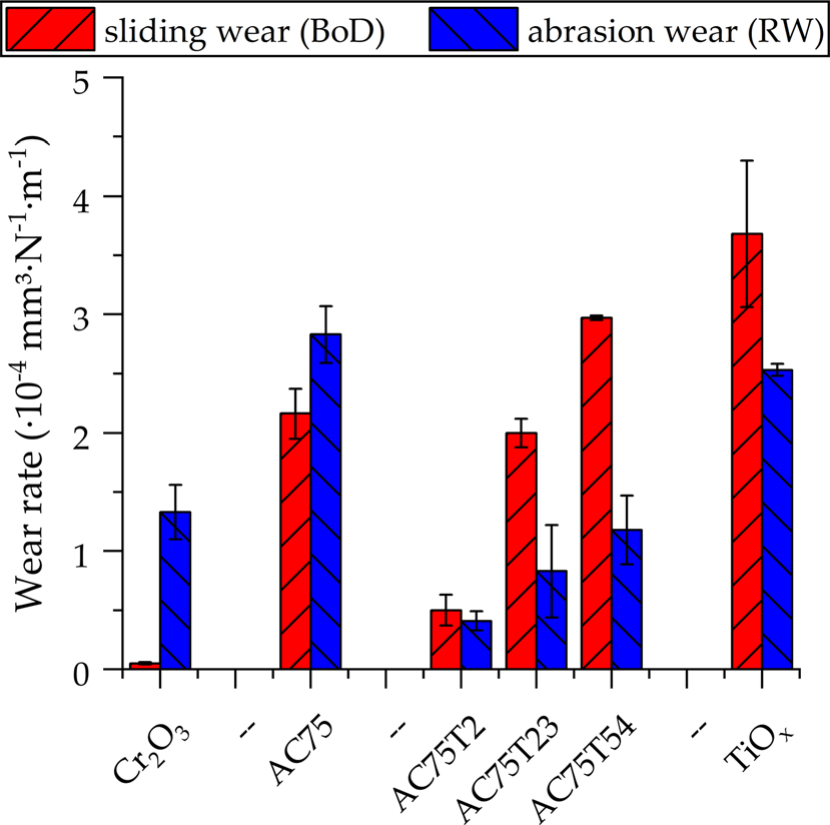
Sliding (ball on disk) and abrasion (rubber wheel) wear rates of the coatings

Figure 9 summarizes the wear rates under sliding and abrasive load. The Cr_2_O_3_ coating showed the lowest sliding wear rate. Both the sliding and abrasive wear rates of the AC75 coating are high, while the coating sprayed from the AC75-T2 powder blend showed a very good resistance in both wear tests. The ranking of the coatings regarding their abrasion wear resistance is different. For all TiO_*x*_-containing coatings and the plain TiO_*x*_ coating abrasion wear rate is lower than the sliding wear rate. For the Cr_2_O_3_ and AC75 coatings, the sliding wear rate is lower than the abrasion wear rate.

For all TiO_*x*_-containing coatings and the plain TiO_*x*_ coating, the abrasion and sliding wear rate increases with the TiO_*x*_ content.

**Fig. 10:**
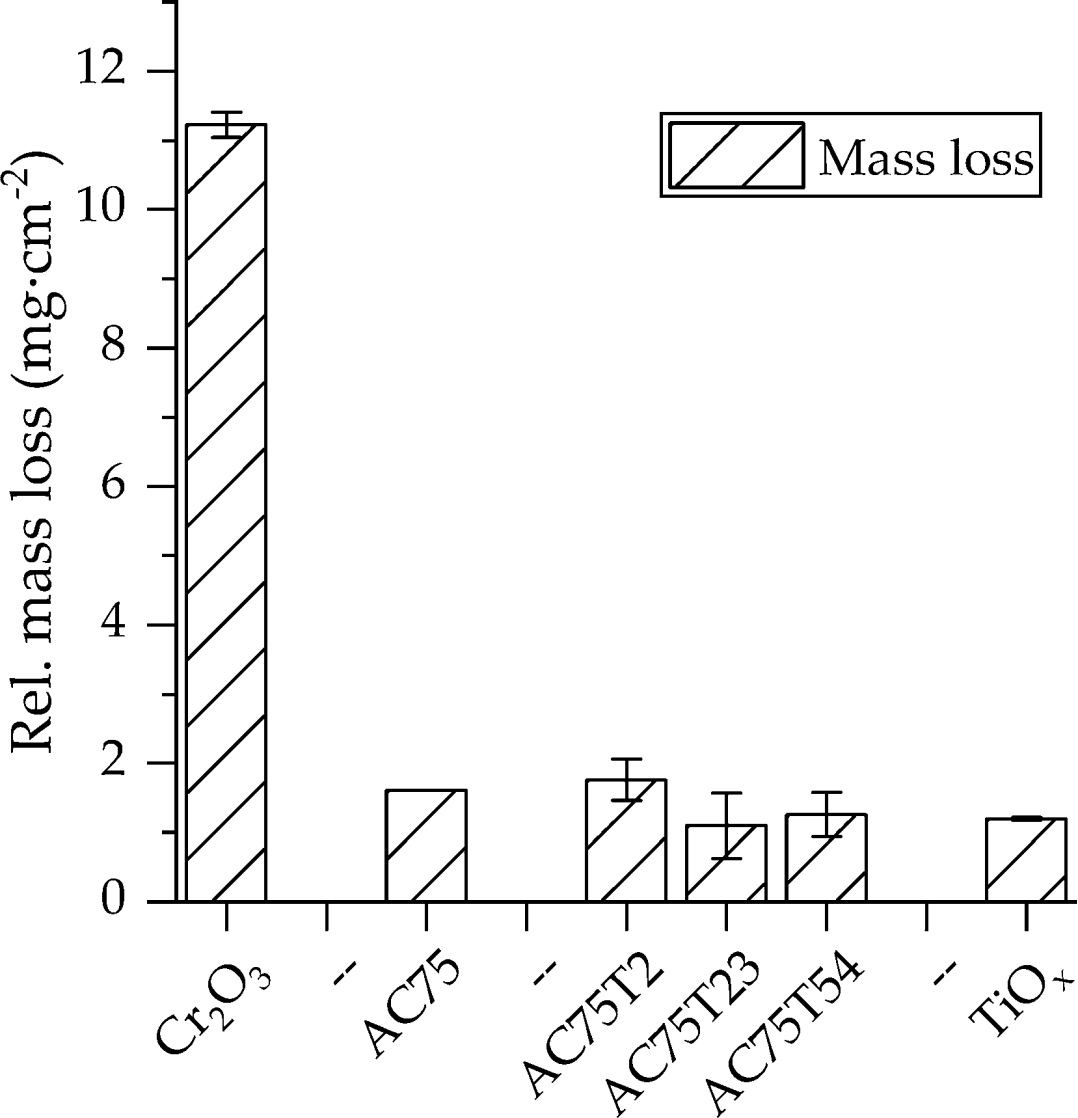
Mass loss of the coatings in 0.5M H_2_SO_4_ for 300 h at 80 °C

The mass loss in the exposure tests of the coatings in 0.5 M H_2_SO_4_ is shown in Fig. 10. They clearly show that the AC75 coating exhibited a significantly higher corrosion resistance than the plain Cr_2_O_3_ coating and reached a similar level as the titanium oxide coating. The coatings sprayed from the powder blends show all comparable values.

**Fig. 11:**
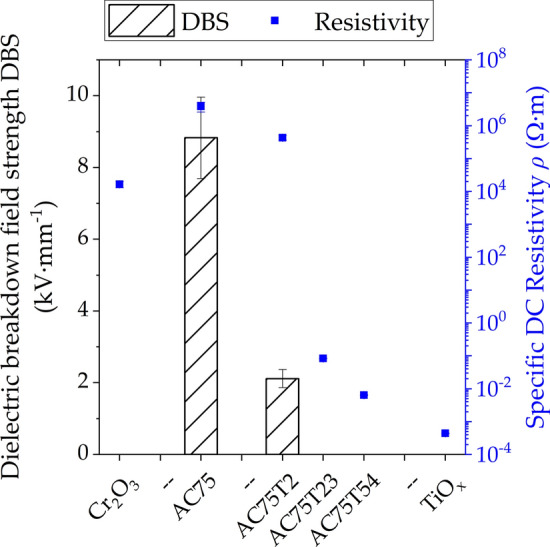
Dielectric strength and specific DC resistivity of the coatings

The electrical properties of the coatings are shown in Fig. 11. The AC75 coating had a higher DC resistivity than the plain Cr_2_O_3_ coating. The small TiO_*x*_ content of 2 wt.% had a minor influence only. However, with a further increasing amount of TiO_*x*_, the electrical resistivity dropped rapidly and became similar to that of the plain titanium oxide coating. The AC75 coating had a dielectric breakdown strength of 8.83 kV/mm. Even small additions of TiO_*x*_ drastically reduce the dielectric strength of the coating. For higher amounts of TiO_*x*_, as well as for plain chromium oxide or titanium oxide coatings, no dielectric strength was measured.

## Discussion

Surprisingly, coatings from commercially available Cr_2_O_3_-rich ss (Al,Cr)_2_O_3_ powders were very rarely described in the literature and are in fact limited to the most recent study of Bolelli et al. (Ref 1), which also used a plain Cr_2_O_3_ coating for comparison. However, the powders used in that study had different compositions, containing 16 wt.% and 35 wt.% Al_2_O_3_. Despite some differences, the spray parameters in that work and in this study are close enough to each other in order to compare the material behavior. Similar to the present study, the deposition efficiency for the ss (Al,Cr)_2_O_3_ solid solution powders was significantly increased compared to the plain Cr_2_O_3_ powder. On the other hand, an increase in porosity was not mentioned in that study. A decrease in coating hardness compared to the Cr_2_O_3_ coating was also observed in that work, but lower than in this study.

In a recent study of the authors (Ref 18), when spraying a ternary blend of fused and crushed Al_2_O_3_, Cr_2_O_3_ and TiO_*x*_ powders by APS, it was mentioned that the deposition efficiency of these individual oxides is strongly dependent on their thermophysical properties and does not depend on the melting temperature only. It was proposed that the "Difficulty of melting factor" (DMF) (Ref 11) and the thermal diffusivity are most suitable to describe their processability. These properties indicated that Al_2_O_3_ is the most difficult material of these three oxides to process, followed by Cr_2_O_3_. The properties of TiO_2_ are strongly deviating, and in addition, the strong decrease in the melting temperature of titanium oxide due to non-stoichiometry has to be taken into account (Ref 18, 21).

The thermophysical data of the ss (Al,Cr)_2_O_3_ used in this study are not known, but it can be assumed that the DMF is higher and thermal diffusivity is lower compared to plain Cr_2_O_3_. It has to be also taken into account that the AC75 powder has some internal porosity due to manufacturing by agglomeration and sintering. Different from dense particles manufactured by fusing and crushing, the internal porosity influences both the heat and momentum transfer from the plasma to the particle (Ref 28). A locally increased Al_2_O_3_ content in the ss (Al,Cr)_2_O_3_ worsened the state of melting of the particles. This is provided by the appearance of the non-molten particles with increased Al_2_O_3_ content in the coating, which mainly show a darker grayscale in the SEM images (e.g., Fig. 5d,f).

In the experiments of this study, a commercial ss (Al,Cr)_2_O_3_ powder was blended with different amounts of a TiO_*x*_ powder, having significantly deviating thermophysical properties. However, the selected spray parameter set assured that the change of the composition of the blends during spraying can be neglected. Blending with TiO_*x*_ resulted in microstructures with less pores and microcracks compared to the AC75 coating (especially cracks between splats). The improved microstructure results in higher hardness values for all compositions. As discussed below, the other properties (with exception of the corrosion resistance) depend strongly on the TiO_*x*_ content.

The homogeneity of the distribution of the metallic elements in the feedstock powder governs the chemical interactions during the spray process. In the current study, Cr_2_O_3_-rich ss (Al,Cr)_2_O_3_ particles are sprayed together with TiO_*x*_ particles. First important point is the stability of the solid solution. Different from Al_2_O_3_-rich ss (Al,Cr)_2_O_3_ particles in previous studies (Ref 9, 10, 16, 29), there is no transformation to *γ*-Al_2_O_3_. However, there is slight change of the composition of the ss (Al,Cr)_2_O_3_ indicating a small loss of chromium, found also when the AC75 powder was blended with TiO_*x*_. This loss could be associated with the formation of volatile chromium oxides. Different from the plain chromia coating, for all (Al,Cr)_2_O_3_-based coatings, no metallic chromium was observed by the SEM and EDS investigations. It should further be mentioned that in the case of spraying blends by DGS (Ref 12) and APS (Ref 13 and 15), for compositions containing 80% Cr_2_O_3_ and 90% Cr_2_O_3_, respectively, no *γ*-Al_2_O_3_ was found in the coatings. In the pattern of the TiO_*x*_, as the (101) and (111) peaks of rutile were observed, while the (110) peak is missing. Thus, it is assumed that in the coating exist non-stoichiometric phases only. In the AC75-T23 and AC75-T54 coatings, small amounts of titanium were detected in ss (Al,Cr)_2_O_3_ splats using EDS point measurements. The content increases with the increasing content of TiO_*x*_ in the powder blend, because there is an increased probability that TiO_*x*_ particles and (Al,Cr)_2_O_3_ particles can interact during the APS process. A small transfer of titanium to other particles was observed also in our previous studies. This included the occurrence of small amounts of Ti in Cr_2_O_3_ (Ref 10, 19), observed also for coatings sprayed from binary suspensions (Ref 30). Different from a study with alumina-rich compositions (Ref 10), where titanium is found in *γ*-Al_2_O_3_, in this study, it is also present in the corundum structure of ss (Al,Cr)_2_O_3_.

Chromium oxide coatings have a superior sliding wear resistance (Ref 1), (Ref 31, 32) within the group of ceramic coatings, which is comparable with that of hardmetal coatings (Ref 31, 32). On the other hand, the abrasion wear resistance of the Cr_2_O_3_ coating among the coatings in the Al_2_O_3_-Cr_2_O_3_ system is known to be low (Ref 33). This corresponds to the results of the current study. Due to the action of the abrasive particles on the porous and microcracked microstructure individual splats of the brittle coatings tend to break off. Compared to the plain Cr_2_O_3_ coating, both sliding and abrasion wear resistance of the AC75 coating are lower, which can also be attributed to the high porosity and low hardness. It should be pointed out that the low hardness measured for the AC75 coatings is most likely due to the cracked microstructure. It can be assumed that the hardness of AC75 coatings can most likely be improved by using a spray parameter set tailored for this individual powder. Remarkably, the hardness values of the AC75-T2 coating are significantly higher than those of the two components. A similar behavior in the sliding and abrasion wear tests was observed and described in detail in a recent work by Bolelli et al. (Ref 1) for various binary Cr_2_O_3_-based systems. Bolelli et al. (Ref 1) stated that the sliding wear rate is governed by the hardness, as the stress is limited to a small area and can therefore be compared with the indentation test as used for determining the hardness. Due to the use of coarse abrasive particles in the rubber wheel test, larger areas are stressed, and the toughness of the coating is more important for the wear behavior of the coating.

By using TiO_*x*_ additions for the AC75 powder, a significantly better abrasion wear performance was obtained. The most balanced coating performance was achieved by blending the ss (Al,Cr)_2_O_3_ with 2 wt.% TiO_*x*_, as this coating showed both high sliding and abrasion wear resistance. With increasing TiO_*x*_ contents, both the abrasion and sliding wear resistance decreased. However, the explanation of the more complex behavior of coatings sprayed from blends requires still more detailed investigations of the mechanical properties.

The chromium oxide coating reveals a moderate corrosion stability between plain alumina coatings (21 mg/cm^2^) (Ref 10) and plain titania coating (1 mg/cm^2^). Remarkably, the (Al,Cr)_2_O_3_ solid solution coating exhibited a corrosion resistance similar to the plain titania coating. Consequently, all coatings sprayed from blends have a similar corrosion resistance as well. The use of solid solution feedstock and blending with TiO_*x*_ might be effective way to improve the corrosion resistance of Cr_2_O_3_ coatings, requiring bondcoats to protect corrosion-sensitive metallic substrates (Ref 34).

The AC75 coating exhibited the highest resistivity and breakdown strength, due to the existence of the (Al,Cr)_2_O_3_ solid solution. A conductive penetrating network is formed with the increasing TiO_*x*_ content, which leads to a fast decrease of the resistivity with increasing TiO_*x*_ content. It can be proposed that the resistivity is adjustable by the TiO_*x*_ content.

The dielectric breakdown strength is only measurable when an electrical field can build up faster than it can be dissipated by the conductivity of the material, as it was the case for the AC75 and AC75-T2 coatings. Both TiO_*x*_ and Cr_2_O_3_ are semiconductors, as according to the definition they have a band gap of 0.1 to 4 eV. Ti_4_O_7_ as a well-known non-stoichiometric phase has a band gap of 0.4 eV (Ref 35) (compared to 3.2 eV for rutile (Ref 36)), while the band gap of Cr_2_O_3_ is 3.3 eV (Ref 37). Thus, the high content of non-stoichiometric titania in AC75-T23 and AC75-T54 is responsible that the dielectric breakdown strength cannot be measured.

## Conclusions

For improvement of APS Cr_2_O_3_ coating solutions ss (Al,Cr)_2_O_3_ feedstock powders were developed and are commercially available. However, these powders and coatings sprayed thereof were only rarely studied in the past. In the current study, further improvement by blending of ss (Al,Cr)_2_O_3_ (AC75) feedstock powder with TiO_*x*_ on the processing properties, as well as on coating microstructure and properties, was investigated. This solid solution feedstock powder showed a high stability, as there is no α- to *γ*-phase phase transformation like for the alumina-rich solid solutions in the Al_2_O_3_-Cr_2_O_3_ system. Only a small reduction of the Cr_2_O_3_ content from 78 wt.% in the powder to 72 wt.% (64 mol%) in the coating resulted from the spray process. Some particles with an increased Al_2_O_3_ content in the ss (Al,Cr)_2_O_3_ showed a poorer melting state, and they occur more frequently as non-molten particles in the coating. The (Al,Cr)_2_O_3_ solid solution coatings already possess some advantages over plain chromium oxide coatings, such as a higher deposition rate or a significantly improved corrosion resistance. However, the high porosity and numerous microcracks have a significant adverse effect on the wear resistance of the AC75 coatings. At this point, it should be mentioned again that all coatings were processed using a uniform parameter set in order to ensure the comparability throughout the different feedstock powders. It is assumed that the microstructure and properties of the AC75 coating can be improved by using a tailored spray parameter set. Many experimental observations made in this study are in full agreement with the results of the recent work by Bolelli et al. (Ref 1), e.g., the opposite ranking of the coatings under sliding and abrasive wear conditions. However, the composition of two ss (Al,Cr)_2_O_3_ feedstock powder grades in their work was different to the one in the present study.

Furthermore, it was intended to improve the properties of the (Al,Cr)_2_O_3_ solid solution coatings by blending with TiO_*x*_, presenting this way a variety of compositions in the ternary Al_2_O_3_-Cr_2_O_3_–TiO_2_ system. Thus, in this work, two types of particles were mixed, where in one type of particles a homogeneous distribution of the metallic constituents (Al and Cr) at an atomic level exists. The third metallic constituent exists separately in the other particle types. The thermophysical properties, such as DMF and thermal diffusivity, of the ss (Al,Cr)_2_O_3_ particles differ significantly from those of the TiO_*x*_ particles. Due to the advantageous thermophysical properties of the latter, the deposition efficiency was increased. Coatings from these blends showed a reduced porosity and defect density (cracks), resulting in an increased coating hardness. Depending on the TiO_*x*_ particle content, the splats formed from the ss (Al,Cr)_2_O_3_ contained some titanium indicating some mass transfer between the individual spray powder particles of ss (Al,Cr)_2_O_3_ and TiO_*x*_ during the spray process. The most balanced coating performance was achieved by blending the ss (Al,Cr)_2_O_3_ with 2 wt.% TiO_*x*_, as this coating showed both a high sliding and abrasion wear resistance, in combination with a high corrosion resistance. Compared to the plain AC75 coating, only the electrical insulation properties were negatively affected. With increasing TiO_*x*_ contents, both the abrasion and sliding wear resistance decreased. However, even at high TiO_*x*_ contents of 54%, an improvement of the abrasion wear resistance compared to plain Cr_2_O_3_ and (Al,Cr)_2_O_3_ coatings was found. The corrosion resistance against H_2_SO_4_ was basically not influenced by the increasing amounts of TiO_*x*_. Only the electrical (insulating) properties are negatively affected by increasing amounts of TiO_*x*_ in the blends.
